# From buckets to backyards: a typology of community-based copepod interventions for *Aedes* vector control—a narrative review

**DOI:** 10.1186/s13071-026-07573-0

**Published:** 2026-07-20

**Authors:** Peter Dambach, Valerie R. Louis, Carlos Alberto Montenegro Quinonez

**Affiliations:** 1 Heidelberg Institute of Global Health (HIGH), Heidelberg University Hospital, Heidelberg, Germany; 2 Facultad de Ciencias Quimicas y Farmacia, Universidad de San Carlos de Guatemala, Guatemala, Guatemala

**Keywords:** Copepods, *Aedes aegypti*, Community-based vector control, Dengue prevention, Implementation models, Narrative review

## Abstract

**Background:**

Biological control using copepods, predatory freshwater crustaceans, has been explored as a strategy for suppressing *Aedes* mosquito larvae in domestic water containers. Although predation efficacy has been documented in laboratory and field settings, less attention has been given to how community-based copepod interventions are organised, delivered, monitored, and sustained under routine conditions. To support operational planning and adaptation, clearer insight is needed into how programmes mobilise communities, produce and distribute copepods, and maintain coverage over time.

**Methods:**

We conducted a narrative, purposive synthesis of published and grey literature, complemented by the authors’ field experience in Asia and Latin America. Sources were considered when they described field-based copepod deployments with sufficient operational detail to inform comparison of production arrangements, distribution channels, community roles, monitoring routines, or replenishment mechanisms. Using iterative cross-case comparison, we identified recurrent implementation configurations and organised them into a provisional typology of delivery models.

**Results:**

Documented approaches include centralised public-health-led programmes, community-facilitated support and rearing systems, household-maintained post-programme approaches, and market-oriented or microenterprise pathways. Across settings, interventions differed in where copepods were produced, who placed them into containers, how communities participated, and how monitoring and replenishment were organised. The evidence base is uneven: centralised public health programmes are the most extensively documented, whereas household-led continuation and market-oriented approaches are less consistently reported and should be interpreted more cautiously. Cost information was limited and difficult to compare across contexts, with total programme costs shaped by staffing, training, monitoring intensity, transport, and facilitation requirements.

**Conclusions:**

This narrative review proposes a heuristic typology for describing and comparing implementation modalities of community-based copepod deployment for *Aedes* vector control. Rather than identifying a single preferred model, the framework clarifies organisational choices that influence operational continuity, including production, last-mile delivery, community engagement, monitoring, and replenishment. Making these delivery architectures explicit can support better reporting, planning, adaptation, and evaluation of copepod-based interventions within integrated vector management.

**Graphical Abstract:**

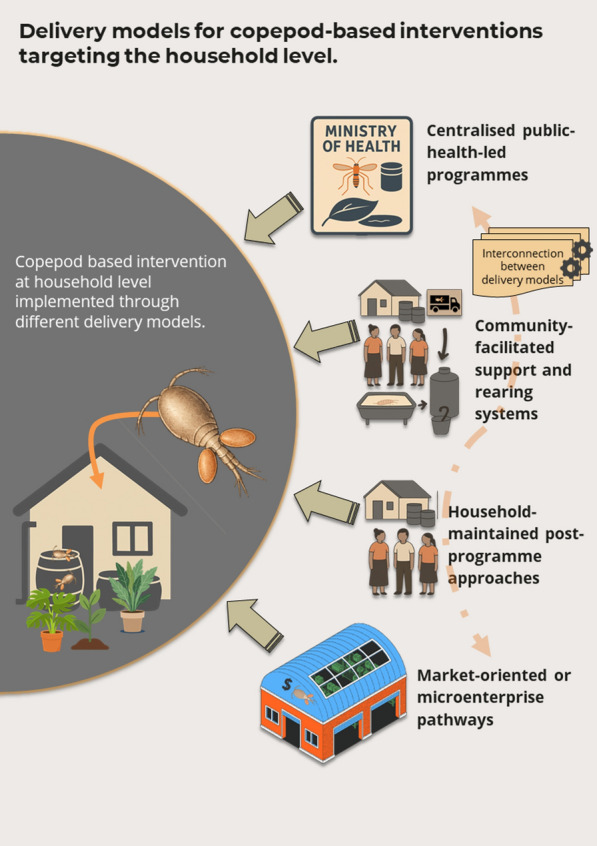

## Background

Dengue, transmitted primarily by *Aedes aegypti*, threatens nearly half of the global population and is expanding in both geographic reach and transmission intensity, driven by rapid urbanisation, changing mobility patterns, and climate change [1–4]. These trends increase the operational difficulty of maintaining effective vector control coverage in domestic and peri-domestic environments, where *Aedes* breeding sites are numerous, dispersed, and frequently recreated through everyday water storage and household practices.

Vector control remains a cornerstone of dengue prevention. However, commonly used approaches such as source reduction, larviciding, and space spraying are often difficult to sustain under routine programme conditions. Domestic containers may be rapidly re-infested, the residual activity of larvicides such as temephos and *Bacillus thuringiensis israelensis* (Bti) varies with formulation, dosage, water turnover, container type, organic matter, and local use practices, and insecticide resistance increasingly constrains chemical options [5–8]. Systematic reviews and evidence syntheses on dengue vector control have similarly highlighted the challenge of achieving durable, community-level impact with conventional tools alone [9].

These limitations have renewed interest in biological and ecological approaches that can complement conventional tools while better aligning with everyday water storage practices [10]. Within the broader logic of Integrated Vector Management (IVM), several environmentally compatible strategies have been explored. Among them, cyclopoid copepods have drawn attention because they can persist in container habitats where *Aedes* commonly breed and prey on early larval stages.

Cyclopoid copepods can be reared locally at comparatively low cost and, under suitable conditions, may maintain predation pressure for weeks to months [11]. Such conditions generally include relatively undisturbed containers, compatible water quality, limited chemical contamination or detergent exposure, sufficient prey availability, and container-use practices that do not repeatedly remove or kill copepod populations. Field implementations across Asia and the Americas have reported reductions in larval or pupal indices in settings where copepods were combined with community engagement, monitoring, and periodic replenishment. The most extensive evidence comes from Vietnam, where multi-year, community-embedded interventions reported reduced *Aedes* indices and, in some settings, epidemiological or serological indicators consistent with reduced dengue transmission risk [12–14]. Importantly, these outcomes were embedded in organised delivery systems, including trained collaborators, scheduled follow-up, and coordinated community or institutional roles.

Despite this potential, the scientific evidence on implementation remains fragmented. Many studies emphasise entomological performance while providing limited or inconsistent detail on programme organisation. Operationally important aspects, including responsibility for inoculation and replenishment, location of rearing, mechanisms for maintaining colony persistence, and the role of local institutions, are not always reported in comparable ways. Recent reviews have called for clearer operational reporting and greater attention to implementation design in dengue vector control and environmental management approaches [9, 15]. However, the copepod literature still lacks a shared framework for describing and comparing delivery arrangements.

This gap matters because field experience suggests that production and delivery architecture is an important operational determinant of whether copepod interventions can be maintained beyond short project periods. Programmes with defined responsibilities, routine monitoring, and feasible replenishment mechanisms appear better able to maintain container coverage than approaches relying solely on irregular effort or household memory. At the same time, biological, ecological, social, and institutional factors all shape outcomes, and no single delivery feature should be understood as sufficient on its own. Without a common implementation vocabulary, practitioners may struggle to adapt copepod interventions to new contexts, and researchers may find it difficult to interpret heterogeneous findings.

In this paper, we address this need by developing a practical, implementation-oriented typology of community-based copepod interventions for *Aedes* vector control. We classify documented field experiences according to four observable operational dimensions: (1) where copepods are reared, (2) who delivers them to containers, (3) how communities participate, and (4) what mechanisms support monitoring and replenishment. These dimensions capture core organisational choices that shape delivery architecture and help distinguish recurrent implementation configurations, including public-health-led, community-facilitated, household-maintained, and market-oriented pathways.

The proposed typology is intended as a heuristic framework rather than a definitive or exhaustive classification. It provides a common language for describing delivery architectures, clarifies responsibilities and replenishment logic, and supports more transparent comparison across settings. By linking biological potential to organisational reality, it offers a planning tool for researchers, implementers, and policymakers seeking to design copepod interventions that are operationally robust, context-appropriate, and aligned with the principles of Integrated Vector Management.

## Methods

This study aimed to develop a practical typology of community-based copepod interventions for *Aedes* control that reflects how such programmes are organised and delivered under field conditions. Because implementation arrangements are described inconsistently and are often embedded within diverse programme reports, we used a narrative, purposive synthesis approach. The review was not designed as a systematic review, did not follow PRISMA procedures, and did not aim to provide exhaustive coverage or quantitative assessment of intervention effectiveness. Instead, the objective was to identify and compare operational patterns relevant to implementation design.

Relevant material was assembled through purposive searches of PubMed, Google Scholar, reference lists of key reviews and primary studies, and selected grey literature or programme documents. Search terms were combined iteratively and included terms such as copepods, *Mesocyclops*, cyclopoid copepods, *Aedes*, dengue, biological control, community-based vector control, implementation, monitoring, and Integrated Vector Management. Additional sources were identified through citation chaining and the authors’ prior field experience with copepod-based vector control in Asia and Latin America.

Sources were considered eligible for the synthesis when they described field-based copepod deployment and provided sufficient operational detail to inform comparison of at least one of the following dimensions: copepod production or rearing arrangements, last-mile delivery channels, community participation structures, monitoring routines, replenishment mechanisms, or sustainability arrangements. Laboratory-only predation studies and purely taxonomic or ecological studies were not central to the synthesis unless they informed operational issues such as species selection, survival, or production feasibility.

For each included field experience, we extracted and summarised operational features across four dimensions: (1) rearing site, describing where copepods were produced and by whom; (2) delivery channel, identifying the actors responsible for introducing copepods into containers; (3) community role, describing the extent and character of local participation; and (4) sustainability and replenishment mechanisms, referring to routines for monitoring, re-inoculation, and continued support. These dimensions were selected because they capture the recurring organisational choices most relevant for maintaining copepod coverage under routine conditions.

Cases were compared iteratively to identify recurrent configurations of delivery arrangements. These configurations were consolidated into four provisional implementation archetypes: centralised public health programmes, community-facilitated support and rearing systems, household-led post-programme maintenance, and market-oriented or microenterprise models. When programmes evolved across phases or combined features of more than one model, classification was based on the dominant delivery structure during the sustained implementation phase, with hybrid or transitional features noted where relevant. The resulting typology should therefore be read as a practical heuristic for implementation analysis rather than as a rigid or exhaustive classification.

### Typology of community-based copepod interventions

**Fig. 1:**
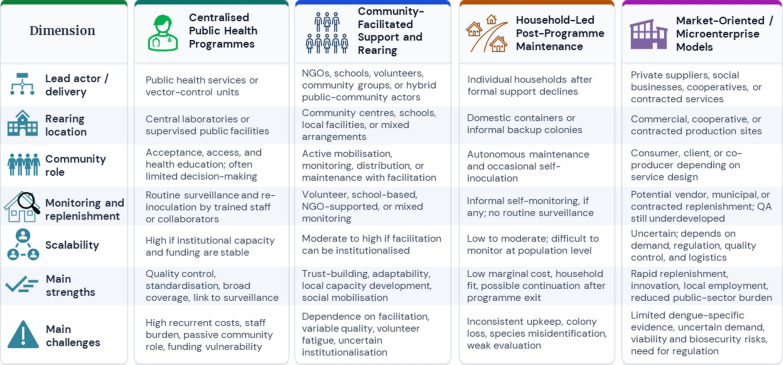
Typology and key implementation dimensions of community-based copepod interventions for Aedes control. The table compares four heuristic implementation models across core operational dimensions. The categories are not mutually exclusive, and the evidence base is strongest for centralised public health programmes and more limited for household-led continuation and market-oriented pathways

Using the operational dimensions described above, we identify four recurrent implementation configurations for community-based copepod deployment: centralised public health programmes, community-facilitated support and rearing systems, household-led post-programme maintenance, and market-oriented or microenterprise models (Fig. 1). These categories are not mutually exclusive, and several programmes have moved between configurations over time. They are best understood as heuristic operational archetypes that highlight differences in lead actors, rearing arrangements, last-mile delivery, community participation, monitoring, and replenishment logic.

The evidence base is uneven across models. Centralised public health programmes, especially those documented in Vietnam, are the most extensively described. Community-facilitated approaches are also represented in the literature, but boundaries between NGO, public-sector, school-based, and volunteer-supported roles are often blurred. Household-led approaches are documented mainly as post-programme carry-over states once formal support has declined. Market-oriented models remain emerging and comparatively underdocumented in dengue vector control, although supply-chain elements and early retail or municipal examples suggest possible future pathways.

### Centralised public health programmes

Centralised public health programmes represent the most structured and best documented model of copepod-based vector control. In this approach, public institutions, typically ministries of health, regional vector control units, or state research centres, assume primary responsibility for copepod production, quality control, distribution, and monitoring. These programmes are characterised by formal hierarchies, standardised procedures, and broad coverage goals. Community participation is present, but often focuses on awareness, acceptance, and household access rather than direct responsibility for rearing or decision-making.

Vietnam's copepod programmes provide the clearest and most influential example of this model. Beginning in the 1990s as part of an integrated strategy to control dengue, the Vietnamese Ministry of Health collaborated with international research groups to launch pilot and later larger-scale interventions in multiple provinces, including Khanh Hoa and Quang Nam. *Mesocyclops thermocyclopoides* and *Mesocyclops aspericornis* were selected based on predatory performance, ecological compatibility, and suitability for cultivation in household water storage jars [12, 14, 16]. Copepods were produced in organised facilities or under supervised conditions, and trained health workers or collaborators supported household distribution, container inoculation, and routine monitoring. Public education campaigns supported uptake by explaining the purpose of copepods and addressing concerns about visible organisms in domestic water containers [13, 14]. In some settings, schoolteachers and pupils contributed to health education and household communication [11].

Evaluations of these programmes reported substantial reductions in larval indices, with Breteau and House indices falling markedly in intervention areas. Some studies also reported serological or epidemiological indicators consistent with reduced dengue transmission risk among children or communities exposed to the interventions [12–14]. For the purpose of this review, however, the key implementation lesson is not the magnitude of entomological impact, but the delivery architecture that enabled sustained coverage: defined institutional responsibility, trained personnel, routine monitoring, and replenishment mechanisms.

Centralised programmes also face important operational constraints. Continuous production, distribution, and household-level monitoring require staffing, transport, training, supervision, and sustained financing. Health workers may already be responsible for multiple public health duties, and copepod handling or entomological surveillance may fall outside routine workflows. The model also depends on political commitment and reliable supply chains, which may fluctuate with funding cycles or leadership changes [10, 17].

A further limitation is that community engagement may remain relatively passive. Households may accept copepods but not acquire the knowledge, motivation, or routines needed to maintain colonies independently. Copepod die-offs caused by container cleaning, chemical contamination, or water turnover may therefore require external re-inoculation. In Vietnam, continued technical support and sustainability funds contributed to ongoing breeding and deployment activities in some areas, but questions remained regarding long-term recurrent costs and institutionalisation [13, 14, 18].

The centralised public health model demonstrates that copepod deployment can be organised at scale when institutional capacity is strong. It is best suited to settings with reliable public health infrastructure, stable funding, and established community access. Its sustainability, however, depends on institutional continuity and on complementary mechanisms that gradually strengthen local ownership and reduce dependence on external re-inoculation.

### Community-facilitated support and rearing systems

The community-facilitated model refers to interventions in which non-state actors, schools, community groups, volunteers, or hybrid public-community arrangements support the adoption and maintenance of copepod-based mosquito control. The defining feature is not necessarily NGO-led rearing, but the presence of an intermediary facilitation layer that provides training, mobilisation, technical support, monitoring, or social reinforcement. These roles may be performed by NGOs, development partners, schools, research teams, municipal actors, or volunteer networks, often in collaboration with public health authorities.

Evidence from Vietnam illustrates how external facilitation can strengthen community ownership within otherwise public-sector programmes. In the Programme Meso-Vietnam (2007–2010), copepods were introduced alongside intensive community education and school-based engagement. The initiative was led by the National Institute of Hygiene and Epidemiology but implemented with support from international development partners, who helped design participatory training and foster networks of local collaborators and teachers to sustain copepod distribution and follow-up [19]. While not a purely NGO-led programme, it demonstrates how facilitation can bridge scientific expertise, public health structures, and community practice.

Longitudinal follow-up of *Mesocyclops* programmes in southern Vietnam further shows the importance of facilitation and social reinforcement. Tran and colleagues [20] examined how responsibility shifted from externally supported projects to local households and documented the fragility of copepod persistence when routine collaborator activities declined. Their findings suggest that training alone may be insufficient unless accompanied by reminders, monitoring, and mechanisms for re-inoculation.

Outside Asia, Monte Verde, Honduras provides an example of integrated biological control supported by community facilitation. The project combined *Mesocyclops* copepods with tilapia, turtles, environmental management, limited larvicide use, volunteer monitoring, and household engagement [21]. It should therefore not be interpreted as evidence of isolated copepod effectiveness. Rather, it illustrates a facilitation architecture in which community engagement, household visits, volunteer monitoring, and external support were combined to organise local vector control. Project accounts indicate that Operacion Bendicion Honduras, a non-profit organisation, helped convene educational meetings, provided facilitators, and supported the volunteer monitoring system [22].

The strengths of community-facilitated approaches lie in their adaptability, trust-building potential, and ability to embed vector control activities in local social structures. Such models may be particularly relevant where public-sector capacity is limited or where community confidence in government programmes is weak. However, the evidence base remains heterogeneous, and facilitation roles are often embedded within broader integrated vector management programmes. It is therefore difficult to isolate the contribution of NGOs or community facilitation from other intervention components.

Community-facilitated models also face sustainability risks. Many depend on short-term project funding, motivated local champions, or volunteer labour. Without a clear transition plan, activities may weaken once external support ends. Their long-term viability depends on whether facilitation functions can be institutionalised through schools, community health workers, municipal services, local associations, or other durable structures.

### Household-led post-programme maintenance

The household-led model is best understood as a post-programme or low-support continuation state rather than as a fully established primary implementation strategy. It emerges when formal public, NGO, or community-facilitated structures withdraw and some households continue to maintain copepods in domestic water containers without routine collaborator visits, organised re-inoculation, or formal monitoring. The defining feature is autonomous domestic maintenance, which distinguishes this model from community-facilitated approaches where a coordinating layer remains active.

Vietnam provides the clearest empirical evidence for this transition. Large community programmes in central and northern Vietnam showed that *Mesocyclops* can contribute to suppression of *Aedes aegypti* when communities are mobilised and collaborator networks periodically re-inoculate key containers and troubleshoot problems [14]. However, when responsibility shifted towards households and routine collaborator support declined, coverage and persistence became more fragile. In southern Vietnam, Tran and colleagues observed that after formal handover to communities and reduced collaborator activity, *Aedes* larval indices increased while *Mesocyclops* prevalence declined; qualitative findings linked this decline to limited household knowledge, loss of reminders and re-inoculation, and reduced surveillance [20]. Kay and colleagues similarly emphasised that the long-term sustainability of *Mesocyclops* programmes depended on periodic collaborator visits and re-inoculation [23].

Other settings reinforce the same operational lesson. In Colombia, a city-scale intervention using *Mesocyclops longisetus* in catch basins maintained low larval densities where basins were visited and managed, but copepod populations failed where basins were exposed to synthetic washing agents [24]. Although this was not a household-run programme, it illustrates a key vulnerability of unsupervised maintenance: routine domestic or environmental practices can eliminate copepod colonies unless there is a mechanism to detect and restock failed sites.

Framed in this way, household-led maintenance occupies a distinct but cautious place in the typology. It shows that copepod use may persist informally in some homes after formal projects end, and it has potential advantages: low marginal cost, alignment with domestic water-storage routines, and reduced dependence on formal delivery systems. At the same time, the literature suggests that population-level operational continuity is difficult to sustain without at least light-touch reinforcement. Risks include uneven technical knowledge, colony loss through cleaning or chemical exposure, species misidentification, lack of quality control, and absence of surveillance data for programme managers.

The household-led model therefore should not be interpreted as evidence that unsupported domestic maintenance is sufficient for sustained vector control. Rather, it highlights what may remain when organised programmes recede and underscores the importance of designing transition mechanisms, reminders, backup colonies, periodic checks, or community-level support structures before formal implementation ends.

### Market-oriented or microenterprise models

The market-oriented model refers to the prospective delivery of predatory cyclopoid copepods as a priced product or contracted replenishment service by private suppliers, social businesses, cooperatives, or hybrid public–private arrangements. Compared with the other models, this pathway remains emerging and comparatively underdocumented within dengue vector control. It should therefore be interpreted as a possible implementation direction rather than a mature, well-established model.

The feasibility of such a pathway rests partly on production knowledge from sectors outside public health. Copepods have long been cultivated as live feed in aquaculture and the ornamental fish trade, creating technical experience in mass culture, packaging, and distribution [25, 26]. What remains less developed is the targeted production and sustained delivery of predator cyclopoids for *Aedes* larval habitats in domestic or peri-domestic settings, including quality assurance, species selection, viability during transport, user guidance, and replenishment systems.

European experiences illustrate some of the supply-chain components that could support market-oriented or hybrid models. In Bologna, Italy, the Centro Agricoltura Ambiente produced cyclopoids centrally and inoculated municipal allotment rain barrels managed by citizens [27]. This example is municipal rather than market-driven and should not be read as direct evidence of a commercial dengue-control model. It is nevertheless relevant as a para-public supply-chain template showing how centrally produced copepods can be distributed across many small domestic-type containers. Direct-to-consumer retail also exists in Italy, where vendors market cyclopoid copepods for household use in rain barrels, tanks, and ornamental containers [28]. In Germany, early private-sector initiatives have begun exploring copepod production and sale for use against *Aedes albopictus* within integrated vector management, although peer-reviewed implementation evidence remains limited.

Outside Europe, route-based municipal stocking provides a logistics template that could, in principle, be adapted to contracted service models. In Cali, Colombia, civic teams stocked street catch basins with *Mesocyclops longisetus* through a standardised many-small-sites distribution architecture [24]. The Cali intervention itself belongs more appropriately under public or civic implementation models; it is referenced here only because its logistics resemble a service route that could be adapted for public–private or contracted replenishment.

Market-oriented pathways could offer several potential advantages, including local entrepreneurship, rapid replenishment services, reduced burden on public health staff, and integration with household-level demand for mosquito control. However, major uncertainties remain. Demand may be unstable, willingness to pay is unknown in many endemic settings, and quality assurance is essential because species identity, predator viability, and ecological compatibility determine operational value. Biosecurity, regulation, user instructions, and monitoring systems would also be required. For these reasons, market-oriented delivery should currently be regarded as an emerging or prospective pathway requiring careful evaluation rather than as an established dengue-control model.

## Discussion

This narrative review proposes a heuristic typology for understanding how community-based copepod interventions for *Aedes* vector control are organised, delivered, and sustained. The central message is not that one model is universally preferable, but that operational architecture matters: copepod-based control depends on biological predation capacity as well as on the systems that produce copepods, place them in containers, monitor their persistence, and replace them when colonies fail. This pattern mirrors broader lessons from dengue vector control and Integrated Vector Management, where organisation of work, community participation, and continuity of implementation often determine whether interventions persist beyond short project periods [9, 30–32].

The strongest and most detailed evidence for sustained copepod implementation comes from Vietnam. In these programmes, regular collaborator rounds, defined responsibilities, training, and predictable replenishment helped maintain low *Aedes* indices over several years and, in some settings, were associated with reduced indicators of dengue transmission risk [12–14, 23]. For this review, the most relevant lesson is that sustained operational coverage was achieved through organised routines rather than through passive household adoption alone. Experiences from Colombia and Honduras similarly suggest that follow-up, monitoring, and community engagement are critical for maintaining copepod populations in the face of container cleaning, detergent exposure, environmental change, or declining household attention [21, 24].

**Fig. 2:**
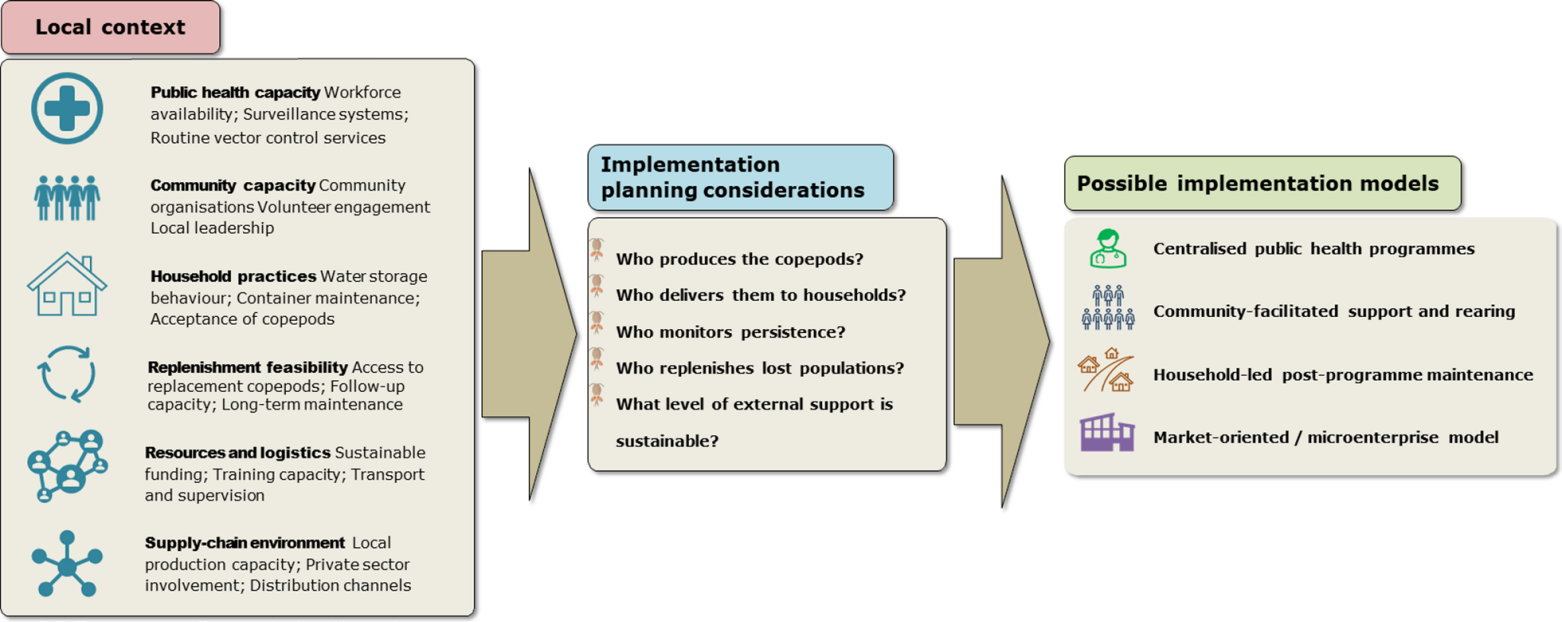
Framework for context-informed selection of copepod implementation models

The typology helps clarify the trade-offs between different implementation configurations (Fig. 2). Centralised public health programmes offer standardisation, quality control, and potential for broad coverage, but they require stable financing, staff time, and institutional commitment [13, 23]. Community-facilitated systems may build trust and local capacity, especially where public-sector reach is limited, but often depend on external facilitation, volunteers, or project-based funding [19, 21]. Household-led post-programme maintenance has low marginal cost and may allow some continuation after formal projects end, but evidence suggests that it is fragile without reminders, re-inoculation, or monitoring [20, 24]. Market-oriented approaches remain emerging and uncertain; they may draw on production techniques from aquaculture and early retail or municipal examples, but require evidence on demand, quality assurance, regulation, and service models before they can be considered established dengue-control pathways [25–27, 28, 29].

Several practical considerations recur across models. First, last-mile responsibility must be explicit. Programmes that specify who places copepods, who checks containers, and who replenishes failed colonies are better positioned to maintain operational coverage than those relying on diffuse household responsibility. Second, training and follow-up are necessary because routine practices such as container cleaning, detergent use, or water replacement can eliminate copepod colonies. Third, linking activities to durable local institutions, including schools, primary health care, municipal services, or community organisations, may reduce the loss of momentum after initial project support ends. Fourth, equity considerations should be addressed explicitly, because water storage, container maintenance, and household vector control responsibilities may fall unevenly across gender, age, and socioeconomic groups, as seen in broader community-based dengue control efforts [30, 31].

Cost evidence remains an important gap. The biological production of copepods can be inexpensive, but total programme costs depend on much more than production. Staffing, supervision, transport, training, quality control, monitoring intensity, community facilitation, and replenishment all shape the cost profile of implementation. Existing cost information is sparse and difficult to compare across settings because programmes vary in scale, institutional arrangements, labour inputs, and integration with existing health systems [23, 25]. Future studies should report operational costs in more standardised ways, including recurrent costs, volunteer time, in-kind contributions, and transition costs when programmes move from externally supported to locally maintained models.

The typology also highlights several knowledge gaps. First, more evidence is needed on transitions between models, particularly how programmes can move from public or project-supported implementation towards sustainable local ownership without losing coverage. Second, reporting of operational details should be improved so that future reviews can compare implementation arrangements more systematically. Third, the long-term feasibility of market-oriented or hybrid public–private delivery remains uncertain and requires careful evaluation of quality assurance, ecological suitability, regulation, affordability, and willingness to pay. Finally, copepod interventions should be evaluated as part of integrated vector management systems rather than as stand-alone tools, since most documented field programmes combine biological control with education, environmental management, larviciding, or other measures.

This review has limitations. Because the synthesis was narrative and purposive rather than systematic, it should not be interpreted as an exhaustive mapping of all copepod interventions. The available literature is uneven, with some programmes described in detail and others reported only briefly or through grey literature. The typology also simplifies complex programmes that may evolve over time or combine features of several models. Nevertheless, this simplification is useful if understood as a heuristic framework for implementation analysis rather than as a rigid classification. By making delivery architecture explicit, the typology can support clearer reporting, better programme design, and more meaningful comparison across settings.

## Conclusions

This narrative review proposes a practical typology for interpreting community-based copepod interventions for *Aedes* vector control through an implementation lens. The framework distinguishes four heuristic delivery configurations: centralised public health programmes, community-facilitated support and rearing systems, household-led post-programme maintenance, and market-oriented or microenterprise models. These models differ in their lead actors, production systems, delivery channels, community roles, monitoring routines, and replenishment mechanisms.

Rather than identifying a single preferred approach, the typology underscores that copepod-based interventions must be matched to local institutional capacity, household practices, ecological conditions, and available resources. The review also shows that the evidence base is uneven: centralised public health programmes are best documented, while household-led continuation and market-oriented pathways require more cautious interpretation and further study. Making implementation architecture explicit can help researchers report operational details more consistently, assist implementers in designing context-appropriate delivery systems, and support policymakers in deciding what level of coordination and monitoring is needed for sustained biological control.

Copepods should therefore be understood as one potential component of integrated vector management. Their public health value depends not only on predation biology but also on the practical systems that keep them present, viable, and accepted in the containers where *Aedes* mosquitoes breed.

## Data Availability

Data supporting the main conclusions of this study are included in the manuscript.
